# Maternal and Fetal Outcomes Among Pregnant Women Exposed to Violence

**DOI:** 10.7759/cureus.44715

**Published:** 2023-09-05

**Authors:** Badriah D Al-Marhabi, Wafaa A Fahim, Nouran E Katooa, Arwa A Al-Nujaydi

**Affiliations:** 1 Faculty of Nursing, King Abdulaziz University, Jeddah, SAU; 2 Nursing Administration, East Jeddah Hospital, Jeddah, SAU

**Keywords:** maternal outcome, risk factor, fetal outcome, pregnancy, violence

## Abstract

Introduction

Domestic violence against women is now widely recognized as a public health issue and a major human rights violation on a global scale. It is a significant risk factor for women's health problems. Pregnancy places a woman under significant physical and psychological pressure, even without additional stressors like abuse. This pressure can have a negative impact on both the mother's and the child's health. This study aims to assess the prevalence of violence among pregnant women and to determine the maternal and fetal outcomes among pregnant women exposed to violence.

Materials and methods

This cross-sectional study was conducted among 347 postpartum women to assess maternal and fetal outcomes among those who were exposed to violence during their pregnancy. A face-to-face interview was done using one tool with three parts to collect the necessary data. Part one included socio-demographic characteristics and reproductive history for participants, part two included safe and validated dates-physical violence victimization scale, and part three included maternal and fetal outcomes.

Result

The findings of this study showed that the prevalence of victimization occurred one to three times (28.8%), while 11.5% of victimization occurred four to nine times, and 2.6% of victimization occurred 10 times or more. Many factors play a role in violence, including family income, husband’s jobs, husband smoking, being forced into marriage, a higher number of children, and parity. Additionally, it was discovered that adverse pregnancy and fetal outcomes include preterm birth (PTB), early onset of labor, low birth weight (LBW), and neonatal admission to the intensive care unit.

Conclusion

The result indicates that violence against pregnant women is at a significant rate. Their findings show that there are several factors that may have caused this percentage. Among the factors that contributed to violence in this study were family income, smoking, husbands’ work, forced marriage, the number of pregnancies, and the number of children. To reduce violence during pregnancy, it is crucial to empower women, especially those without a source of income of their own. It is also critical to educate partners and foster healthy relationships between partners.

## Introduction

Domestic violence (DV) against women is now widely recognized as a public health issue and a major human rights violation on a global scale. It is a significant risk factor for women's health problems, having many further implications for both their physical and mental well-being [1]. Around the world, violence affects over one-third (27%) of women between the ages of 15 and 49 [2]. The prevalence of any kind of intimate partner violence (IPV) during pregnancy was 25.0% worldwide. Rates varied by region, with Africa having the highest rate (36.1%) and Europe having the lowest rate (5.1%) [3]. Violence currently has a significant impact on neonatal health outcomes such as low birth weight (LBW), preterm birth (PTB), stillbirth, and pregnancy outcomes such as abortion, hypertension, and post-partum hemorrhage [2].

Lockdowns during the COVID-19 pandemic and because of its economic and social consequences, women are more exposed to violent partners and other established risk factors [4]. The United States (US) indicates that domestic violence instances have increased since the COVID-19 outbreak began. In Oregon, US, domestic abuse hotlines saw an increase in demand for their services as COVID-19 spreads throughout the state [5]. DV can manifest in a variety of ways, including sexual (sexual assault and degrading behavior), physical (injuring, incapacitating, and in its most extreme form, killing the victim), emotional (constant, unrelenting verbal barrage of insults and criticisms), and psychological (indignity, controlling what the victim can and cannot do, embarrassing the victim, isolation from friends or family, and financial) forms [6].

According to a study conducted in Canada by Muldoon et al., one in four pregnant women was exposed to violence from a partner [7]. In the study done in Nigeria, the lifetime prevalence rates of DV among women were between 33.1% and 63.2%, and prevalence rates among pregnant women ranged from 2.3% to 44.6% [8]. Also, a study by Priya et al. reported that about 60% of the Indian women who were screened were positive for DV [9]. A cross-sectional study was conducted in Minya Governorate, Egypt, on 513 pregnant women, which showed that 50.8% were cases exposed to violence during pregnancy, and the prevalence of physical, sexual, verbal, and emotional was 30.2%, 20%, 41.7%, and 45.4%, respectively [6]. Additionally, in Saudi Arabia, a study conducted on 400 currently married females aged 19-65 years found that the prevalence of IPV was 44.8%, in the form of physical (18.5%), emotional (25.5%), sexual (19.2%), and economic (25.3%) violence [10].

Pregnant victims of violence often describe being choked, scalded, forced out of moving cars, punched, kicked, shoved downstairs, threatened with knives, and having things thrown at them. As a result of the abuse they endure, individuals also suffer from a variety of violence-related ailments, such as cuts, bruises, fractures, concussions, tooth injuries, knife wounds, vaginal bleeding, and chronic migraines [11].

The complex and dynamic interaction of social, cultural, political, and psychological elements leads to DV. The following elements are significantly associated with experiences of violence, even though none of these characteristics alone can fully explain why violence occurs. Women’s occupation, family income, partners’ alcohol use, women’s responsibility to be pregnant, history of being exposed to child abuse, seeing family violence, and having an antisocial personality disorder, all remained significant predictors of DV during pregnancy [6,10].

In Vietnam, a prospective cohort study of 1276 pregnant women in Dong Anh district found that PTB or LBW was statistically significantly associated with maternal exposure to physical abuse during pregnancy. Pregnant women who experienced physical abuse during their pregnancies had a five-time increased risk of developing PTB and a nearly six-time increased risk of giving birth to LBW children [12]. In Iran, physical, sexual, and emotional domestic violence were found to be, respectively, 16.4%, 18.6%, and 44.4% among 528 pregnant women, and the most reported complications of violence included bleeding, preterm labor, and hypertension [13].

A study done in China by Yu et al. also discovered a strong correlation between PTB and LBW. Also, in Ethiopia, PTB and LBW are five times more common in pregnant women who experienced violence [1]. Additionally, a study in Saudi Arabia discovered a potential increase in preterm birth for pregnant assault victims [14]. This study aims to assess the prevalence of violence among pregnant women and to determine the maternal and fetal outcomes among pregnant women exposed to violence.

## Materials and methods

Study design, area, population, and sampling

An exploratory descriptive cross-sectional design was carried out in the post-partum department at East Jeddah Hospital, Saudi Arabia, over six months from September 2022 to February 2023 on post-partum women after obtaining ethical approval from the Faculty of Nursing College at King Abdulaziz University issued, and the research ethics committee of the education department of East Jeddah Hospital region for the application of the study. The data was collected through a survey. The aim of the study was explained to the study participants on the cover page. A convenience sample of 347 postpartum women who were available at the postpartum unit in the hospital. The inclusion criteria were post-partum women aged 18 to less than or equal to 45 years old, post-partum or post-abortion women, Arabic speakers, and willingness to participate in the study. The excluded criteria were postpartum women who have a history of mental disorders according to hospital records and non-Arabic speakers.

Instrument

One tool to collect necessary data includes three parts, as follows: Part one: This part includes two sections and was developed and used by the researcher to collect the necessary data; it entailed the following sections; Section one: included socio-demographic data of pregnant women such as age, level of education, occupation, marital status, living, age of husband and level of education, husband’s job, family income, smoking, and alcohol intake for husband; Section two: included the reproductive history of pregnant women such as gravidity, parity, number of abortions, number of living children, type of last delivery, and the problem during intercourse facing the pregnant woman and her husband.

Part two: Safe and validated dates-physical violence victimization scale: It was developed by Foshee 1998 and Linder and Bauman 1996, which measures the women's physical abuse experienced in her relationship with her husband within the last year. It consists of 15 questions. Each question is rated on a four-point scale ranging from zero to three according to the degree she experiences violence; zero: no physical victimization, one: physical victimization occurs one to three times, two: physical victimization occurs four to nine times, three: physical victimization occurs 10 and more time. The final score is calculated as the sum of all 15 questions. A high score means great abuse [15].

Part three: The maternal and fetal outcomes, in this part, questions regarding the pregnant women's maternal and fetal outcomes were: Section one: The maternal outcome includes: abortion, the onset of labor, time of childbirth, type of delivery (normal, assisted, and cesarean sections), presence of signs of maternal distress, mode of rupture of membranes (spontaneous or artificial), time of rupture of membranes (mature or premature), duration of each stage of labor, complications occur during each stage of labor; Section two: fetal outcome includes: birth weight, height, head, and chest circumferences, fetal death, and admission to the neonatal intensive care unit.

Data analysis

The collected data were coded, categorized, and tabulated using proper statistical significance tests to determine the relationship between the variables using SPSS statistics for Windows version 25.0 (IBM Corp., Armonk, NY, USA). Frequencies and percentages were used to summarize data and texts and tables were used to present data. Also, the Chi-square test was used to investigate the relationship between two categorical variables. The significance was stated as p≤0.05 as the level of significance.

## Results

A total of 347 post-partum women participated in the current study. The women participants had different socio-demographic characteristics. Those who were aged less than 25 years old constituted 15.9% of the study participants, while about one-third was 31-35 years old. However, more than one-third of the participant's husbands were aged 36-45 years old. While a significant proportion of pregnant women with a university education (35.7%). Most of the participants live in their husband’s houses (83.8%) (Table 1). On the other hand, most husbands with secondary education are 51.3%. Also, most participants were housewives (73.5%), and their husbands had a job (Table 2).

**Table 1:** Distributed the study sample according to socio-demographic characteristics of women (n=347).

Variables		Frequency	Percentage
Age	Less than 25	55	15.9%
	25-30 years	90	25.9%
	31-35 years	117	33.7%
	More than 36 years	85	24.5%
Educational level	Illiterate	18	5.2%
	Less than secondary	88	25.4%
	Secondary school	117	33.7%
	University	124	35.7%
Occupation	Working	64	18.4%
	Housewife	255	73.5%
	Student	28	8.1%
Living	A separate house for the husband	291	83.8 %
	With the husband's family	41	11.8%
	With the wife's family	4	1.2%
	A separate house for the wife	11	3.2%
Family income	It is enough	180	51.9%
	Increases	22	6.3%
	Barely enough	113	32.6%
	Not enough	32	9.2%

**Table 2:** Distributed the study sample according to the socio-demographic characteristics of the husband (n=347).

Variables		Frequencies	Percentage
Age of husband	Less than 25	3	0.9%
	25-30 years	54	15.6%
	31-35 years	123	35.4%
	36-45 years	126	36.3%
	More than 45 years	41	11.8%
The husband’s educational level:	Illiterate	15	4.3%
	Less than secondary	65	18.7%
	Secondary school	178	51.3%
	University and more	89	25.7%
Husband's job	Working	266	76.7%
	Does not work	31	8.9%
	Retired from work	15	4.3%
	Free business	35	10.1%
Does the husband smoke?	Yes	168	48.4%
	No	179	51.6%
Does he take any drugs or alcoholic beverages?	Yes	6	1.7%
	No	341	98.3%

Most of the participants in the study had between two and seven years of marriage, and a small proportion of women were forced into marriage; also, 11.0% of their husbands had second wives (Table 3).

**Table 3:** Distributed the study sample according to marital characteristics (n=347).

Variables		Frequencies	Percentages
Years of marriage	Up to one year	45	13.0%
	2-7 years	166	47.8%
	8-12 years	91	26.2%
	More than 13 years	45	13.0%
Are you forced into marriage?	Yes	42	12.1%
	No	305	87.9%
Are you a first wife?	Yes	246	70.9%
	No	101	29.1%
Is your husband married to another woman?	Yes	38	11.0%
	No	309	89.0%
If yes, the number of wives (n=38)	One	15	39.5%
	Two	20	52.6%
	Three	3	7.9%
Consanguinity	Yes	164	47.3%
	No	183	52.7%

Most of the women participating in the study had experienced three to four pregnancies. In addition, the highest percentage hadn’t experienced abortion, and the last delivery was normal. A smaller percentage of participants and their husbands had a problem during intercourse (Table 4).

**Table 4:** Distributed the study sample according to reproductive history (n=347).

Variables		Frequencies	Percentages
Gravidity	One time	56	16.1%
	Two times	90	25.9%
	Three to four times	132	38.1%
	Five times and more	69	19.9%
Parity	Non	91	26.3%
	One to two time	101	29.3%
	Three to four times	103	29.8%
	Five times and more	50	14.6%
The number of abortions	Non	232	66.9%
	One to two times	97	28.0%
	Three to four times	13	3.7%
	Five times and more	5	1.4%
Number of living children	Non	93	26.8%
	One to two times	104	30.0%
	Three times	86	24.8%
	Four times and more	64	18.4%
Type of last birth (n=254)	Normal delivery	174	68.5%
	Cesarean section	65	25.6%
	Using assistive devices	15	5.9%

The prevalence of violence was reported to be 42.9% (Table 5). The level of prevalence of violence against pregnant women was assessed through a questionnaire of safe statements-victim of physical violence scale. The findings of this study showed that the prevalence of victimization occurred one to three times (28.8%), while (11.5%) victimization occurred four to nine times, and (2.6%) victimization occurred 10 times and more.

**Table 5:** Distribution of the participants according to the violence score (n=347).

Variables	Frequencies	Percentages
No physical victimization	198	57.1%
Victimization occurred one to three times	100	28.8%
Victimization occurred four to nine times	40	11.5%
Victimization occurred ten times and more	9	2.6%

There is a significant relationship between a score of violence and other variables such as family income, smoking, husbands’ work, forced marriage, the number of pregnancies, and the number of children. This study recounted that there was a significant relationship between victimization and family income. About 52.3% of pregnant women exposed to violence are in a family with barely enough income, and 62.6% are exposed to violence with not enough income (Table 6). Also, this current study found a significant relationship between victimization and the husband’s job. Around 38% of pregnant women whose husbands have jobs and 51.5% whose husbands have free businesses were exposed to violence compared to pregnant women whose husbands have not worked and retired from work (Table 7). Pregnant women whose husbands smoked were exposed to violence 52.4% compared with those whose husbands did not smoke (Table 7). While half of the participants of pregnant women who were forced into marriage reported being exposed to violence (Table 8). Also, the study found a significant relationship between victimization and the number of parties. Pregnant women who had one to two times parity were exposed to violence by 48.5%, and 46% were exposed to violence who had five times or more. The study found pregnant women who have one to two children exposed to violence by 45.2% and 36% who have three children (Table 9). However, the study found that 66.7% of pregnant women have problems during intercourse exposed to violence (Table 10). 

**Table 6:** The relationship between socio-demographic characteristics of women and exposure to violence.

		Victimization								Chi-square	Sig.
		No physical victimization		Victimization occurred one to three times		Victimization occurred four to nine times		Victimization occurred 10 times and more			
		Count	%	Count	%	Count	%	Count	%		
Age	Less than 25 years old	31	56.4%	13	23.6%	8	14.5%	3	5.5%	14.401	0.109
	25–30 years old	44	48.9%	35	38.9%	10	11.1%	1	1.1%		
	33–35 years old	76	65.0%	31	26.5%	8	6.8%	2	1.7%		
	More than 36 years old	47	55.3%	21	24.7%	14	16.5%	3	3.5%		
Educational level	Illiterate	8	44.4%	5	27.8%	3	16.7%	2	11.1%	13.788	0.130
	Less than secondary	44	50.0%	26	29.5%	14	15.9%	4	4.5%		
	Secondary school	70	59.8%	36	30.8%	9	7.7%	2	1.7%		
	University and more	76	61.3%	33	26.6%	14	11.3%	1	0.8%		
Occupation	Working	30	46.9%	21	32.8%	10	15.6%	3	4.7%	6.438	0.376
	Housewife	148	58.0%	73	28.6%	28	11.0%	6	2.4%		
	Student	20	71.4%	6	21.4%	2	7.1%	0	0.0%		
Living	A separate house for the husband	174	59.8%	79	27.1%	33	11.3%	5	1.7%	15.525	0.077
	With the husband’s family	17	41.5%	14	34.1%	7	17.1%	3	7.3%		
	With the wife’s family	3	75.0%	1	25.0%	0	0.0%	0	0.0%		
	A separate house for the wife	4	36.4%	6	54.5%	0	0.0%	1	9.1%		
	Another	0	0.0%	0	0.0%	0	0.0%	0	0.0%		
Family income	It is enough	119	66.1%	49	27.2%	10	5.6%	2	1.1%	44.047	0.000*
	Increases	13	59.1%	4	18.2%	5	22.7%	0	0.0%		
	Barely enough	54	47.8%	41	36.3%	15	13.3%	3	2.7%		
	Not enough	12	37.5%	6	18.8%	10	31.3%	4	12.5%		

**Table 7:** The relationship between socio-demographic characteristics of the husband and exposure to violence

		Victimization								Chi-square	Sig.
		No physical victimization		Victimization occurred one to three times		Victimization occurred four to nine times		Victimization occurred 10 times and more			
		Count	%	Count	%	Count	%	Count	%		
Age of husband	Less than 25 years old	3	100.0%	0	0.0%	0	0.0%	0	0.0%	10.348	0.586
	25–30 years old	32	59.3%	15	27.8%	5	9.3%	2	3.7%		
	33–35 years old	72	58.5%	38	30.9%	11	8.9%	2	1.6%		
	36–45 years old	71	56.3%	31	24.6%	19	15.1%	5	4.0%		
	More than 45 years old	20	48.8%	16	39.0%	5	12.2%	0	0.0%		
The husband’s educational level:	Illiterate	9	60.0%	4	26.7%	1	6.7%	1	6.7%	5.576	0.782
	Less than secondary school	33	50.8%	20	30.8%	10	15.4%	2	3.1%		
	Secondary school	99	55.6%	55	30.9%	19	10.7%	5	2.8%		
	University and more	57	64.0%	21	23.6%	10	11.2%	1	1.1%		
Husband’s job	Working	165	62.0%	71	26.7%	25	9.4%	5	1.9%	25.589	0.002*
	Does not work	8	25.8%	11	35.5%	9	29.0%	3	9.7%		
	Retired from work	8	53.3%	6	40.0%	1	6.7%	0	0.0%		
	Free business	17	48.6%	12	34.3%	5	14.3%	1	2.9%		
Does the husband smoke?	Yes	80	47.6%	54	32.1%	26	15.5%	8	4.8%	16.645	0.001*
	No	118	65.9%	46	25.7%	14	7.8%	1	0.6%		
Does he take any drugs or alcoholic beverages?	Yes	4	66.7%	0	0.0%	1	16.7%	1	16.7%	6.660	0.084
	No	194	56.9%	100	29.3%	39	11.4%	8	2.3%		

**Table 8:** The relationship between marital characteristics and exposure to violence.

			Victimization								Chi-square	Sig.
			No physical victimization		Victimization occurred one to three times		Victimization occurred four to nine times		Victimization occurred 10 times and more			
			Count	%	Count	%	Count	%	Count	%		
Years of marriage		Up to one year	24	53.3%	10	22.2%	9	20.0%	2	4.4%	11.991	0.214
		Two to seven years	90	54.2%	55	33.1%	17	10.2%	4	2.4%		
		8–12 years	59	64.8%	23	25.3%	6	6.6%	3	3.3%		
		More than 13 years	25	55.6%	12	26.7%	8	17.8%	0	0.0%		
Are you forced into marriage?	Yes		21	50.0%	9	21.4%	9	21.4%	3	7.1%	9.199	0.027^*^
	No		177	58.0%	91	29.8%	31	10.2%	6	2.0%		
Are you a first wife?	Yes		138	56.1%	73	29.7%	30	12.2%	5	2.0%	1.705	0.636
	No		60	59.4%	27	26.7%	10	9.9%	4	4.0%		
Is your husband married to another woman?	Yes		15	39.5%	16	42.1%	5	13.2%	2	5.3%	6.198	0.102
	No		183	59.2%	84	27.2%	35	11.3%	7	2.3%		
If yes, the number of wives	One		7	46.7%	5	33.3%	3	20.0%	0	0.0%	4.718	0.580
	Two		6	30.0%	10	50.0%	2	10.0%	2	10.0%		
	Three		2	66.7%	1	33.3%	0	0.0%	0	0.0%		
Consanguinity	Yes		87	53.0%	52	31.7%	21	12.8%	4	2.4%	2.247	0.523
	No		111	60.7%	48	26.2%	19	10.4%	5	2.7%		

**Table 9:** The relationship between reproductive history and exposure to violence.

		Victimization								Chi-square	Sig.
		No physical victimization		Victimization occurred one to three times		Victimization occurred four to nine times		Victimization occurred 10 times and more			
		Count	%	Count	%	Count	%	Count	%		
Number of pregnancies- gravidity	One time	36	64.3%	12	21.4%	7	12.5%	1	1.8%	16.833	0.051
	Two times	45	50.0%	30	33.3%	13	14.4%	2	2.2%		
	Three to four times	81	61.4%	42	31.8%	6	4.5%	3	2.3%		
	Five times and more	36	52.2%	16	23.2%	14	20.3%	3	4.3%		
Number of birth-parity	Non	51	54.8%	25	26.9%	16	17.2%	1	1.1%	20.804	0.014*
	One to two times	52	51.5%	36	35.6%	9	8.9%	4	4.0%		
	Three to four times	68	66.0%	29	28.2%	4	3.9%	2	1.9%		
	Five times and more	27	54.0%	10	20.0%	11	22.0%	2	4.0%		
The number of abortions	Non	140	60.3%	66	28.4%	22	9.5%	4	1.7%	9.611	0.383
	One to two times	51	52.6%	27	27.8%	15	15.5%	4	4.1%		
	Three to four times	6	46.2%	4	30.8%	2	15.4%	1	7.7%		
	Five times and more	1	20.0%	3	60.0%	1	20.0%	0	0.0%		
Number of children	Non	51	54.8%	25	26.9%	16	17.2%	1	1.1%	19.246	0.023*
	One to two children’s	57	54.8%	33	31.7%	9	8.7%	5	4.8%		
	Three children’s	55	64.0%	27	31.4%	2	2.3%	2	2.3%		
	Four children’s and more	35	54.7%	15	23.4%	13	20.3%	1	1.6%		
Type of last birth	Normal	104	59.8%	51	29.3%	14	8.0%	5	2.9%	7.054	0.316
	Cesarean section	37	56.9%	19	29.2%	6	9.2%	3	4.6%		
	Using assistive devices	6	40.0%	5	33.3%	4	26.7%	0	0.0%		

**Table 10:** The relationship between the presence of sexual problems during intercourse and exposure to violence.

		Victimization								Chi-square	Sig.
		No physical victimization		Victimization occurred one to three times		Victimization occurred four to nine times		Victimization occurred 10 times and more			
		Count	%	Count	%	Count	%	Count	%		
Does your husband have problems during intercourse	Yes	11	45.8%	4	16.7%	7	29.2%	2	8.3%	12.125	0.007*
	No	187	57.9%	96	29.7%	33	10.2%	7	2.2%		
Do you have problems during intercourse?	Yes	12	33.3%	14	38.9%	6	16.7%	4	11.1%	17.532	0.001*
	No	186	59.8%	86	27.7%	34	10.9%	5	1.6%		

Assessment of adverse maternal outcomes showed that 58.8% had preterm labor and around 53.8% had early onset of labor and labor after the specified date (Table 11). The most common adverse fetal outcomes were LBW (58.2%, Table 12) and 56.7% of neonatal admissions to the intensive care unit whose mothers were exposed to violence (Table 13).

**Table 11:** The relationship between maternal outcomes and exposure to violence.

Variables		Victimization								Chi-square	Sig.
		No physical victimization		Victimization occurred one to three times		Victimization occurred four to nine times		Victimization occurred 10 times and more			
		Count	%	Count	%	Count	%	Count	%		
Abortion	Yes	38	53.5%	20	28.2%	12	16.9%	1	1.4%	2.929	0.403
	No	160	58.0%	80	29.0%	28	10.1%	8	2.9%		
Type of childbirth	Normal	78	58.2%	42	31.3%	13	9.7%	1	0.7%	7.654	0.569
	Induction of labor	26	63.4%	10	24.4%	3	7.3%	2	4.9%		
	Cesarean section	47	54.0%	25	28.7%	10	11.5%	5	5.7%		
	Birth using assistive devices	9	64.3%	3	21.4%	2	14.3%	0	0.0%		
Time of childbirth	Early	14	41.2%	14	41.2%	6	17.6%	0	0.0%	15.991	0.014*
	On time	126	62.4%	55	27.2%	17	8.4%	4	2.0%		
	After the specified date	20	50.0%	11	27.5%	5	12.5%	4	10.0%		
Onset of labor	Early	12	46.2%	11	42.3%	2	7.7%	1	3.8%	14.363	0.026*
	On-time	94	66.2%	33	23.2%	14	9.9%	1	0.7%		
	After the specified date	7	33.3%	11	52.4%	2	9.5%	1	4.8%		
Presence of signs of prenatal distress	Yes	29	54.7%	15	28.3%	7	13.2%	2	3.8%	3.600	0.308
	No	84	61.8%	40	29.4%	11	8.1%	1	0.7%		
Is there an increase in the number of heartbeats?	Yes	9	75.0%	3	25.0%	0	0.0%	0	0.0%	3.860	0.277
	No	20	48.8%	12	29.3%	7	17.1%	2	4.9%		
Is there a decrease in the number of heartbeats?	Yes	1	16.7%	4	66.7%	1	16.7%	0	0.0%	5.633	0.131
	No	28	59.6%	11	23.4%	6	12.8%	2	4.3%		
Did you have hypertension?	Yes	15	46.9%	10	31.3%	5	15.6%	2	6.3%	2.826	0.419
	No	14	66.7%	5	23.8%	2	9.5%	0	0.0%		
Did you have hypotension?	Yes	9	69.2%	3	23.1%	1	7.7%	0	0.0%	1.876	0.599
	No	20	50.0%	12	30.0%	6	15.0%	2	5.0%		
Method of rupture of the membrane before childbirth	Normal	95	59.0%	47	29.2%	16	9.9%	3	1.9%	0.832	0.842
	Un normal	18	64.3%	8	28.6%	2	7.1%	0	0.0%		
The time of rupture of the membrane before birth	Early	22	47.8%	17	37.0%	5	10.9%	2	4.3%	5.779	0.123
	On time	91	63.6%	38	26.6%	13	9.1%	1	0.7%		

**Table 12:** The relationship between fetal outcome when birth and exposure to violence.

Variables		Victimization								Chi-square	Sig.
		No physical victimization		Victimization occurred one to three times		Victimization occurred four to nine times		Victimization occurred 10 times and more			
		Count	%	Count	%	Count	%	Count	%		
Weight at birth	Normal	120	64.2%	47	25.1%	16	8.6%	4	2.1%	13.563	0.035*
	Less than his/her age	23	41.8%	19	34.5%	10	18.2%	3	5.5%		
	More than his/her age	17	50.0%	14	41.2%	2	5.9%	1	2.9%		
Head circumference	Bigger than normal	9	64.3%	3	21.4%	2	14.3%	0	0.0%	7.831	0.251
	Normal	138	59.7%	65	28.1%	20	8.7%	8	3.5%		
	Less than normal	13	41.9%	12	38.7%	6	19.4%	0	0.0%		
The length	Bigger than normal	5	29.4%	7	41.2%	4	23.5%	1	5.9%	11.167	0.083
	Normal	154	59.9%	73	28.4%	23	8.9%	7	2.7%		
	Less than normal	1	50.0%	0	0.0%	1	50.0%	0	0.0%		
Chest circumference	Bigger than normal	6	27.3%	8	36.4%	7	31.8%	1	4.5%	16.168	0.013*
	Normal	151	60.4%	71	28.4%	21	8.4%	7	2.8%		
	Less than normal	3	75.0%	1	25.0%	0	0.0%	0	0.0%		

**Table 13:** The relationship between status after birth and exposure to violence.

Variables		Victimization								Chi-square	Sig.
		No physical victimization		Victimization occurred one to three times		Victimization occurred four to nine times		Victimization occurred 10 times and more			
		Count	%	Count	%	Count	%	Count	%		
The death of the fetus	Yes	38	50.7%	21	28.0%	14	18.7%	2	2.7%	4.940	0.176
	No	160	58.8%	79	29.0%	26	9.6%	7	2.6%		
Admission to the intensive care unit	Yes	26	43.3%	18	30.0%	13	21.7%	3	5.0%	14.061	0.003^*^
	No	134	62.0%	62	28.7%	15	6.9%	5	2.3%		

## Discussion

A total of 347 pregnant women participated in the current study. The results of the current study showed that almost one-half of the study participants were exposed to physical violence during the current pregnancy. This dreadful result exceeds the reported results worldwide. This result is more than a study conducted in Wuhan, China, which found around 18.35% of women exposed to violence during pregnancy [16]. Also, a study by Habib conducted at Ayub Teaching Hospital and Benazir Bhutto Shaheed Teaching Hospital, Abbottabad, from January 2015 to December 2016 found that 35% of 1000 pregnant women were exposed to violence [17].

The difference between the result of the current study and other studies is due to the fact that the current study was carried out with the COVID-19 pandemic consequences, and during any pandemic or epidemic leading to domestic violence increased. Also, consequently, disruption of the social protective networks and increased economic handshape could be the leading cause for increasing the violence against women in the current study. Other studies have been done far from the COVID-19 period [18,19].

A cross-sectional study conducted in Ethiopia by Yohannes in 2019 indicated that 64.6% of pregnant women were exposed to violence in their lifetime [20]. Further, a study conducted among pregnant women in Iran during the COVID period found the prevalence of abuse to be 93.1% [21]. However, in Northern California, researchers showed a 38% rise in the first month of the pandemic [22]. In addition, a study in Canada by Muldoon in 2021 found that one in four women endures violence [7]. A study conducted in Saudi Arabia by Taifi in 2021 reported that around 44.8% of pregnant women were exposed to violence [23].

The findings of this study showed that the prevalence of victimization occurred one to three times (28.8%), while (11.5%) victimization occurred four to nine times, and (2.6%) victimization occurred 10 times and more.

It is crucial to have a firm understanding of both the prevalence of violence as a whole and the key factors that are connected to its occurrence in order to develop intervention approaches. As a result, the current study also made an effort to assess the factors related to the presence of violence. The study recounted that there was a significant relationship between victimization and financial status, while a high percentage of pregnant women were at risk of violence in a family with low or insufficient income. This result agrees with the result of the study by Jatta and Mohler-Kuo, which found an association between low financial status and increased violence [24,25]. However, most participants in the current study were housewives who were less financially empowered and dependent on their husbands for financial support and most of them had suffered physical abuse.

In addition, this current study found a significant relationship between victimization and the husband’s job, a high proportion of the women whose husbands work are exposed to physical violence. This finding is similar to other study findings in Ethiopia [20]. The probability of women being abused by their husbands is likely increased by the stress of the job, the pressure of business, and the stress of trade in the private sector.

This study demonstrates an association between husbands' smoking and physical violence among pregnant women. A study in Gambia and Turkey found that, compared to husbands who never smoke, a smoking husband is significantly related to several types of violence [25,26]. The reason could be that smokers suffer from psychological symptoms like depression and anxiety more than non-smokers due to the presence of nicotine [27].

This current study showed the significant relationship between victimization and pregnant women who were forced into marriage, while half of the participants of pregnant women who were forced into marriage reported being exposed to violence. However, forced marriage is a kind of gender-based violence that increases vulnerability to abuse, particularly domestic violence by husbands [28]. In addition, a higher number of children and parity are associated with a high percentage of physical violence during pregnancy. Similar to other studies conducted in Saudi Arabia by Taifi and Debono [23,29]. An increase in the number of children causes financial hardship and limits women's productive capacity, leading to either an increase or the beginning of physical violence during pregnancy.

The finding of the current study increases physical violence among pregnant women who have problems during intercourse. This fact is due to pregnant women having physiological changes during pregnancy and loss of interest in sex, especially during the first and third trimesters. A study by Khalesi found the female body is forced to start using muscles rarely used before pregnancy because of an increase in both belly volume and fetal weight, which might result in lumbar discomfort [30]. Additionally, pregnancy tends to make sexual relationships less appealing for women because of weariness, worry, and the natural fear they have as labor approaches as well as the non-erotic influence of the woman's appearance near the end of pregnancy, are other factors that contribute to the decline in female sexual function [31].

This current study found that physical violence against pregnant women was consistently associated with a significant increase in adverse birth effects. It was observed that women who had been exposed to violence had a PTB, early onset of labor, LBW, and admission to the intensive care unit. A study in Ethiopia showed that abused pregnant women experience five times more LBW and PTB [1]. Also, a study in Saudi Arabia found a possibility of increases in PTB for abused pregnant women [14]. In addition, a study conducted in China found a significant association between PTB and LBW [16]. In Vietnam, there is an increased risk of PTB and LBW for pregnant women who are exposed to violence [12]. Also, according to a study conducted in Ghana's northern region, mothers who had been subjected to prenatal violence had a threefold increased risk of giving delivery to a child that was underweight and a twofold increased risk of giving delivery prematurely [32].

The relationship between physical violence during pregnancy exposure and fetal outcomes can be explained by various reasonable scientific and psychosocial theories. The physiological responses to stress caused by physical or psychological abuse might affect neonatal outcomes by generating prostaglandin, which can premature contractions and delivery [33]. There are additional explanations for the link between prenatal violence, PTB, and LBW, including the fact that abused women experience high levels of stress and poor mental health, which can result in poor nutrition habits and their consequences, including anemia, underweight, and poor gestational weight gain, which may cause LBW [33,34].

To reduce violence during pregnancy, it is crucial to empower women, especially those without a source of income of their own. It is also critical to educate partners and foster healthy relationships between partners. Additionally, counseling can assist women who have experienced domestic violence in addressing the psychological and emotional effects of the violence. It may be provided to individuals, groups, or couples. Also, safety planning is creating an individualized plan to assist women in identifying and addressing possible risks associated with domestic violence. Creating a safety network of dependable friends, relatives, or advocates, as well as establishing a plan to exit an abusive circumstance, are some examples of what this could entail [35]. However, women who have experienced violence may benefit from legal support and obtaining legal protection, like restraining orders. It may also comprise support for divorce processes and custody. On the other hand, healthcare professionals should do routine follow-ups. To ensure the safety and well-being of pregnant patients who have experienced violence [36].

Limitation

In this study, cross-sectional and self-reported data were obtained because, in Arabic countries, it is a hidden problem; most of them prefer to remain silent to keep their home and family, and they don't want to report exposure to violence to avoid blame from family and community because it will lead to divorce and the loss of their family and home.

## Conclusions

The result indicates that violence against pregnant women is at a significant rate. Further findings show that there are several factors that may have caused this percentage. The factors that contributed to violence in this study were family income, smoking, husbands’ work, forced marriage, the number of pregnancies, and the number of children. To reduce violence during pregnancy, it is crucial to empower women, especially those without a source of income of their own. It is also critical to educate partners and foster healthy relationships between partners.
